# Telemedicine in an adolescent and young adult medicine clinic: a mixed methods study

**DOI:** 10.1186/s12913-023-09634-x

**Published:** 2023-06-22

**Authors:** Angela Barney, Sabrina Mendez-Contreras, Nancy K. Hills, Sara M. Buckelew, Marissa Raymond-Flesch

**Affiliations:** 1 Division of Adolescent and Young Adult Medicine, Department of Pediatrics, University of California, San Francisco, United States; 2 School of Medicine, University of California, San Francisco, United States; 3 Department of Neurology, UCSF Weill Institute for Neurosciences, University of California San Francisco School of Medicine, San Francisco, United States; 4 Philip R. Lee Institute for Health Policy Studies, University of California, San Francisco, United States; 5 Division of Adolescent and Young Adult Medicine, Department of Pediatrics, University of California San Diego, San Diego, United States

**Keywords:** Telehealth, Telemedicine, Adolescent, Young adult, Parent, Confidentiality, Mixed methods, Adolescent medicine

## Abstract

**Background:**

Adolescents and young adults are a diverse patient population with unique healthcare needs including sensitive and confidential services. Many clinics serving this population began offering telemedicine during the Covid-19 pandemic. Little is known regarding patient and parent experiences accessing these services via telemedicine.

**Methods:**

To assess for trends and disparities in telemedicine utilization in the first year of the pandemic, we used the electronic health record to obtain patient demographic data from an adolescent and young adult medicine clinic in a large urban academic institution. Characteristics of patients who had accessed telemedicine were compared to those who were only seen in person. Mean age was compared using t-test, while other demographic variables were compared using chi-squared test or Fisher’s exact test. We performed qualitative semi-structured interviews with patients and parents of patients in order to characterize their experiences and preferences related to accessing adolescent medicine services via telemedicine compared to in-person care.

**Results:**

Patients that identified as female, white race, Hispanic/Latinx ethnicity were more likely to have utilized telemedicine. Telemedicine use was also more prevalent among patients who were privately insured and who live farther from the clinic. Although interview participants acknowledged the convenience of telemedicine and its ability to improve access to care for people with geographic or transportation barriers, many expressed preferences for in-person visits. This was based on desire for face-to-face interactions with their providers, and perception of decreased patient and parent engagement in telemedicine visits compared to in-person visits. Participants also expressed concern that telemedicine does not afford as much confidentiality for patients.

**Conclusions:**

More work is needed to address patient and parent preferences for telemedicine as an adjunct modality to in-person adolescent and young adult medicine services. Optimizing quality and access to telemedicine for this patient population can improve overall healthcare for this patient population.

**Supplementary Information:**

The online version contains supplementary material available at 10.1186/s12913-023-09634-x.

## Background

The Covid-19 pandemic led to rapid implementation of telemedicine services, including at clinics serving adolescent and young adult (AYA) patients [1–3]. AYAs are a developmentally diverse patient population with unique healthcare needs, such as access to sensitive mental and reproductive health services. They require opportunities for confidential alone-time with their clinicians, in addition to ongoing parental engagement in care when appropriate [4]. There is increasing demand for AYA healthcare services, particularly for mental health concerns [5, 6], and telemedicine can play a role in expanding access [7, 8].

The potential impact of digital health technology (which includes social media, mobile health [mHealth], wearable and digital devices, and games for health) on AYA health and privacy has been well studied [9], but study of these these technologies typically does not include telemedicine (audiovisual medical visits).

Previous research has described socioeconomic, racial/ethnic, and geographic disparities in telemedicine use in adult and pediatric populations before and during the pandemic [10–14]. Issues including digital literacy, access to devices and high-speed internet, and health system-created barriers are thought to contribute to these disparities [10, 12], However, it is not clear if these disparities and contributing factors are applicable to the AYA patient population specifically.

In March of 2020, when the first shelter-in-place orders were being implemented in the United States, the University of California San Francisco Adolescent and Young Adult Medicine clinic rapidly changed our clinical practice [1]. Prior to this time, the clinic did not offer telemedicine visits. By the end of March, almost all visits were being conducted via telemedicine [1]. Since then, the clinic has re-opened more in-person visits but continues to offer telemedicine for AYA sub-specialty and primary care.

Prior to the pandemic, there was little evidence regarding the practice of AYA-specific telemedicine. In the first year of the pandemic, some studies found that telemedicine was generally acceptable to AYAs and their families and feasible for conducting AYA medical visits [15, 16]. However, with the pandemic waning, and telemedicine services becoming less compulsory there is a need for better understanding of the nuances of adolescent patient and parent experiences and preferences related to utilizing telemedicine compared to in-person care as we move into the post-pandemic era.

In this mixed methods cross-sectional study we aim to describe the UCSF Adolescent and Young Adult medicine clinic patient population’s access to telemedicine and in-person visits during the Covid-19 pandemic, as well as patient and parent perceptions and experiences with utilizing these services.

## Methods

UCSF’s Adolescent and Young Adult Medicine Clinic is embedded in an urban academic medical center. The clinic was established in 1974. In 2019, the clinic served 1,715 unique 12 to 26-year-old patients through 4,195 clinic visits. The clinic provides full-spectrum interdisciplinary AYA care including primary care for AYAs with complex medical conditions, management of attention and mood disorders, sexual and reproductive health care, eating disorder care, and substance use disorders.

### Statistical analysis

Demographic characteristics of study participants were summarized in total and compared between those who had only attended office visits and those who had attended any video visit. Age was normally distributed and summarized using the mean and standard deviation. All other characteristics were categorical and were summarized with frequencies and percentages. Age was compared between the two groups using a t-test, while categorical variables were compared using chi-squared tests, except when cells had fewer than five observations, in which case the Fisher’s exact test was used.

### Qualitative interviews with patients and parents

The research team is made up of women, one of whom was a clinical fellow in Adolescent and Young Adult Medicine at the time. Two of the researchers are physicians and faculty in the Division of Adolescent and Young Adult Medicine at UCSF. Other researchers included a medical student, and Professor with joint appointments in the UCSF Department of Neurology and Department of Biostatiscs and Epidemiology.

Working within a modified grounded theory framework, we carried out semi-structured interviews with patients and parents of patients from the clinic to characterize their experiences and preferences related to telemedicine. The research team developed interview guides specific to patients or parents, and whether or not they had experience with telemedicine. Interview guides were pilot tested in practice sessions among the researchers. Eligible adolescent and young adult participants were at least 13 years of age, fluent in English or Spanish, and had a clinic visit (either in-person, telemedicine visit, or both) between March 2020 (when the clinic began offering telehealth services) and the time of recruitment. Parents were eligible if they had at least one child who met these criteria.

The research team recruited participants through convenience sampling, using flyers posted in the clinic and individual providers shared information about the study during telemedicine visits. Potential participants who expressed interest while a research assistant was in the clinic were able to meet with them to review study logistics and schedule a time for the interview. 52 potential participants expressed interest, but due to scheduling conflicts or loss of follow-up after the initial contact, 22 of them elected not to participate. The team obtained informed written consent from all adult participants, and informed assent in addition to written parental permission to participate for all minor participants. Interviews were conducted by two of the authors (AB and SM-C). Interviewers were trained by the senior author (MR-F), who is an experienced qualitative researcher, through practice interviews and role play. Interviewers were initially observed conducting interviews by senior author to ensure consistency between interviewers and fidelity to the interview guide. Although AB may have provided direct care in the AYA clinic to some of the participants, she did not conduct the interviews for those participants. At the beginning of the interviews, interviewers explained to participants that as clinicians and researchers interested in AYA health, we wanted to better understand how telemedicine could be useful and what preferences patients and families have for accessing care. The research team includes three clinicians in the AYA clinic, who may have had preconceived notions about what patients and families may think about telemedicine, or our own biases related to the modality having used it extensively throughout the pandemic.

We conducted interviews between July-December, 2021. 20 patients and 10 parents of patients of the UCSF Adolescent and Young Adult Medicine Clinic participated in 60 min in-depth interviews via secure video conferencing (Zoom Video Communications Inc.) or telephone in the language of their choice. Interviews were recorded using the video conferencing platform. Interviewers may have taken notes to guide the interview, but these notes were not included in later data analysis. Only participants and researchers were present during the interviews. No repeat or follow-up interviews were conducted. Interviews were securely recorded and professionally transcribed. One of the interviewers is a native Spanish speaker and conducted the Spanish-language interviews, which were professionally translated and transcribed to English for analysis. Transcripts were not shared or returned to participants. Study participants received gift cards (value of $100 for adult participants and $50 for minors) as compensation.

Interviews focused on patients’ and parents’ experiences accessing telemedicine, barriers to using telemedicine, the impact of telemedicine on patient/provider relationship, and confidentiality. At the end of each interview, the interviewer collected participant demographic information via open-ended questions. Two authors completed iterative rounds of thematic identification and related memos, with themes reviewed and refined by all the authors. Some of the themes identified were anticipated in the interview guide, while others were only identified upon data analysis. Interviews were conducted until data saturation was reached. Participants were not asked to provide feedback on the findings. The UCSF Institutional Review Board approved this study protocol.

## Results

The clinic patient population consisted of 1,651 patients, with 2,684 unique patient encounters from March 2019 through February 2021. The mean age of these patients was 18.1 years (SD 3.2 years), and 69.9% identify as female, 28% as male, with the remainder identifying as nonbinary/gender queer, transgender, or chose not to disclose their gender identity (Table 1).

**Table 1:** Clinic patients who utilized telemedicine compared to patients that were only seen in person March 2019-February 2021

	Total		Telemedicine Visit		**Only Office Visit**		p-value*
	**n = 1651 (%)**		**n = 825 (%)**		n = 826 (%)		
Age in years, mean	18.1	(SD 3.2)	17.7	(SD 3.2)	18.5	(SD 3.2)	< 0.001†
Legal sex							< 0.001**
Female	1186	(71.8)	634	(76.8)	552	(66.8)	
Male	465	(28.2)	191	(23.2)	274	(33.2)	
Gender identity							0.01**
Female	1154	(69.9)	613	(74.3)	541	(65.5)	
Male	462	(28)	186	(22.5)	276	(33.4)	
Nonbinary/Gender Queer	25	(1.5)	20	(2.4)	5	(0.6)	
Transgender Female	2	(0.1)	2	(0.2)	0	(0)	
Transgender Male	3	(0.2)	1	(0.1)	2	(0.2)	
Other/Chose not to disclose	5	(0.3)	3	(0.4)	2	(0.2)	
Race							< 0.001
White or Caucasian	746	(45.2)	396	(48)	350	(42.4)	
Black/African American	178	(10.8)	58	(7)	120	(14.5)	
Asian	255	(15.4)	107	(13)	148	(17.9)	
American Indian/Alaska Native	13	(0.8)	10	(1.2)	3	(0.4)	
Hawaiian/Pacific Islander	9	(0.5)	4	(0.5)	5	(0.6)	
Other	370	(22.4)	186	(22.5)	184	(22.3)	
Unknown/Declined	80	(4.8)	64	(7.8)	16	(1.9)	
Ethnicity							< 0.001
Hispanic/Latino	317	(19.2)	165	(20)	152	(18.4)	
non-Hispanic/non-Latino	1253	(75.9)	596	(72.2)	657	(79.5)	
Unknown/Declined	81	(4.9)	64	(7.8)	17	(2.1)	
Insurance type							0.003
Private	1157	(70.1)	606	(73.5)	551	(66.7)	
Public	494	(29.9)	219	(26.5)	275	(33.3)	
SF county							< 0.001
No	693	(42)	438	(53.1)	255	(30.9)	
Yes	958	(58)	387	(46.9)	571	(69.2)	
Bay area county							< 0.001
No	114	(6.9)	93	(11.3)	21	(2.5)	
Yes	1537	(93.1)	732	(88.7)	805	(97.6)	

* All p-values calculated using chi-square tests, unless otherwise indicated.

** P-value calculated using Fisher’s exact test.

† P-value calculated using t-test.

58% live in San Francisco County, and 93.1% live in counties that make up the Bay Area (San Francisco, Alameda, Contra Costa, Marin, Napa, San Mateo, Santa Clara, Solano, and Sonoma counties) (Table 1). See Table 1 for further details of patient population characteristics.

20 clinic patients ages 13–25 (11 minors, nine young adults) and 10 parents of patients participated in interviews. 23 of the participants identified as female, 13 identified as white, six identified as Hispanic/Latinx (Table 2).

**Table 2:** Interview participant demographics

	Parents	Patients
**Total (n)**	10	20
**Age Range**		
13–17 years	-	11
18 + years	-	9
**Gender**		
Male	1	5
Female	9	14
Other	0	1
**Race/Ethnicity**		
White	4	9
Hispanic/Latinx	3	3
Asian	2	3
Native American	1	1
Multiracial	0	4
Type Of Visits*		
Telemedicine only	2	1
In-person only	3	7
Both	5	12
**Home Location**		
In SF	5	15
Outside SF	5	5
**Language**		
English	7	20
Spanish	3	0

* Type of visits utilized by patient with the clinic, as of time of enrollment

Most of the participants had completed at least one telemedicine visit with our clinic, although 10 of the participants had not utilized telemedicine and had only been seen in the clinic in person (Table 2). 20 of the study participants lived in San Francisco County (Table 2). Most reported English as their preferred language, although three participants (all parents) preferred Spanish and completed the interviews in Spanish (Table 2).

### Quantitative results

Among our clinic’s entire patient population, we found differences in patient demographics among those who had accessed telemedicine compared to those who had only accessed in-person care. There were significant associations between telemedicine utilization and each of the patient demographic variables assessed. Higher proportions of female patients (both by legal sex (p < 0.001) and gender identity (p = 0.01)), white patients (p < 0.001), Hispanic/Latinx patients (p < 0.001), privately insured patients (p = 0.003), patients living outside of the county (p < 0.001) and outside of the major metropolitan area (p < 0.001) utilized at least one telemedicine visit (Table 1). Higher proportions of males, black or Asian patients, and publicly insured patients received only in-person care (Table 1).

### Qualitative results

Study participants generally agreed that telemedicine includes more barriers to privacy than in-person visits. For telemedicine visits taking place in the home, it was possible for family to overhear patients’ private conversations with their providers. One parent shared that “with video visits, I think the temptation to listen in (on patient’s alone time) is too much, so for that aspect I would prefer in person.” One young adult patient reported choosing to join telemedicine visits from their parked car, as this felt more private to them than being in the home. The concern that their conversations can be overheard can limit what patients disclose to their providers during telemedicine visits:

I think it did kind of limit what I said…which probably impacted how the visit went. I really don’t want my parents hearing that. I had some questions, like sexual questions, and yeah, I felt like I kind of had to censor myself a little bit,

- young adult patient.

However, for some patients, telemedicine provided opportunities for privacy and independence that they could not access with in-person appointments. Patients shared that video visits meant that they could attend a medical appointment without being seen by anyone they knew in the waiting room. Patients and parents also acknowledged that telemedicine made it possible for children to access care without their parents knowing.

My mom being there makes me feel less inclined to be honest and open (on intake forms/questionnaires). So sometimes I’ll change the facts a little bit, like on the form, ‘cause I would fill out the form…but then my mom would take it and read the form and look it over, to check what I said, and that always made me feel anxious… I feel like on my screen, on my computer, my parents wouldn’t look at it,

-young adult patient.

Several parents also commented that telemedicine could help their children practice the transition to seeking care on their own and begin to independently manage their healthcare. A parent of a 14-year-old liked that their child could access a telemedicine visit on their own if needed, even though they were not yet independent enough to navigate the public transportation needed to get to clinic in person. The parent valued that telemedicine would allow their child to avoid the safety risks of arranging transportation and have visits on their own much earlier on in adolescence if needed. Even if they personally preferred in-person appointments, all participants acknowledged that telemedicine can improve access to care. The convenience of telemedicine was particularly attractive to one parent who was relieved they would not need to make a trip to clinic every 3 months for their child’s ADHD medication. Telemedicine required less time, and it spared their child the stress of coming into the clinic environment. Similarly, participants identified that telemedicine requires less logistical planning and can help alleviate geographic and financial barriers to care. As one parent shared,

We are at least six hours away and the trek is not an easy trek…We would not have followed up…[Telemedicine] really saved the day…How I would think about it is always against the backdrop of, we have no (adolescent medicine) care at all, where we are. And, you know, it’s always against, you know, (video visits) or no care at all.

Overall, most parents and patients were not concerned about the cyber security of telemedicine visits. However, a few participants did share that they have a general expectation that any information they put on the internet could potentially be accessed by a third party. As one patient shared, “I personally wouldn’t care (about showing physical exam findings on telemedicine) but other people probably would…because people won’t shut up about how anything that is recorded it will be there forever. Digital footprint and stuff…” This patient then shared their misunderstanding that all telemedicine visits are recorded.

Patient and parent perceptions of the effect of telemedicine on their relationships with their providers varied, but most felt that they connected better in-person and were able to have more meaningful interactions. One young adult patient shared that, “in person, it’s just easier to talk…it’s just more human, there’s more connections personally…Teenagers, we have a lot of private things to say, and it’s just easier to say it in person.” Another young adult noted, “Sometimes people do just need a pat on the back or something. You can’t get that through Zoom.” Patients and parents also noted the value of better nonverbal communication in person compared to telemedicine.

I need to have that human one-on-one connection and actually be able to see them and hear them and have all of the nonverbal communication as well… that is really important and that’s why I feel more comfortable sharing (during in-person visits). So, I prefer all of my visits in-person.

-young adult patient

In addition to feeling more connected to providers, patients and parents generally feel more engaged in person.

I think probably like virtually it was hard to stay focused. I know for me and, and my mother too, if we’re physically there then we’re kind of forced to listen.

-adolescent patient

Participants identified more potential distractions on telemedicine, which ranged from noise in the home to distractions on the internet. Some patients disliked that their parents were distracted by other activities or responsibilities during telemedicine visits (joining telemedicine visits remotely from their place of employment, the car, or even the golf course). One young adult patient shared that for telemedicine visits their parents may be, “less involved and maybe not involved at all because, well, I think the reason that they’re usually there (in person) is because they need to drive me there.” Additionally, some parents expressed concern that their children did not take telemedicine visits as seriously and that in-person visits were more impactful. This trend of decreased engagement and accountability was particularly noted by patients who disclosed being treated for eating disorders and their parents. One parent said, “Her weight was sliding a little bit. So that’s the reason why starting in January, I say, ‘Okay, we need to go in person to have the weight of the doctor’s word telling her, you know, she needs to eat.’ We saw (the doctor in person) and I saw a change in her attitude after that.”

Patients and parents also perceived telemedicine visits as having a lower priority or being easier to forget: “Me and my mom we actually did make an appointment… it was on Zoom but we forgot about it. I think also having it on zoom, you can always forget about the times,” one young adult patient shared. AYAs may face additional barriers when joining telemedicine visits on their own. Some participants noted that it can be harder for AYAs to access care without the structured time away from school and parental support required to physically travel to the clinic. As one patient shared: “I have a friend and she had (virtual) therapy every week, and she would just forget. Like, no one would like come and get her to go to therapy so she would literally just forget, and not go…and she didn’t do anything about it… I think she quit.”

On the other hand, some participants reported feeling intimidated by the clinic setting and felt it was easier to open up in a telemedicine visit. They reported that the video visit format felt more distant or informal, making it easier to be open about sensitive topics. One adolescent said, *“*I liked being able to go to my appointments at home, I felt like it was more comfortable…that made it easier to talk with the doctors. I felt like it was kind of easier just because I was in a less anxiety producing environment when I was at home.” For patients who face prejudice and discrimination when accessing medical care, telemedicine could even provide a sense of safety. One transgender patient shared,

I have been discriminated against by doctors in the past, living in Georgia as a transgender kid. I’ve had not great interactions with doctors where they’ve just straight up, made me feel bad about my body. Knowing that there’s another option to not have to experience that, like there’s not gonna be a physical sense of like, this person in my space is making me feel like my body isn’t okay. Which is a matter of safety to a certain extent, especially as a child. So, I think having a virtual platform where you don’t have to fear that your body is not being validated in a physical space is really nice for sure.

-young adult patient

Additionally, some patients and parents liked that telemedicine appointments felt less rushed, and they perceived having more time to ask all their questions.

Despite the rapid movement to telemedicine during the pandemic, very few patients or parents reported experiencing technical issues. Those who did experience issues reported that those challenges made it more difficult to connect emotionally with the provider and made appointments longer than they needed to be. As one adolescent patient shared, “It’s hard whenever you’re trying to have a serious conversation, and it gets broken up by the connection.” One factor that particularly affected the number of technical difficulties patients or their parents experienced was the type of device they used for their appointment. Those who used phones as opposed to computers reported experiencing more technical difficulties. These technical difficulties were compounded by language barriers for Spanish-speaking parents. All the Spanish-speaking parents in our sample only had access to a phone for telemedicine and relied on their children to help them navigate the video software.

## Discussion

This study found that AYA patients and their parents had nuanced perceptions regarding telemedicine, with many expressing preferences for in-person healthcare visits. Although participants acknowledged telemedicine’s convenience and potential for improving access to care, there was a strong preference from some to have face-to-face interactions with clinicians, citing better therapeutic alliance and overall communication during in-person visits. This finding was surprising, particularly for AYA patients, whom we expected to have a strong preference for telemedicine visits given their digital nativism and ubiquitous role of technology in their lives [17].

When we conducted the interviews for this study, many people had become accustomed to using video chat platforms, including for virtual schooling during the pandemic. While we expected this would lend to a comfort with video chat that would positively impact patient and parent perspectives, some participants described “Zoom fatigue,” or general dislike for virtual interactions that gave them a further appreciation for in-person clinic visits. While Zoom fatigue has been described and some studies have measured it among health care providers, little research has explored the phenomenon in patient populations or its impact on the quality of telemedicine visits [18–20]. This issue may be particularly relevant to younger populations who continue to engage in distance learning or in adults who telecommute.

Many participants also expressed concern regarding confidentiality of alone time for AYA patients during telemedicine visits. These findings differ from earlier studies examining the acceptability and quality of telemedicine for AYAs, which found that patients and caregivers viewed telemedicine as highly acceptable and were satisfied overall with confidentiality of telemedicine visits [15, 16]. Participants’ concerns regarding confidentiality of patient “alone time” were notable, particularly given that both patients and parents expressed similar concerns. Assurance of confidentiality has been well documented to improve disclosure of sensitive health information for adolescent patients [21]. In this study participants expressed concerns that confidentiality is not always possible in the home environment, and some patients gave specific examples of how their concern of being overheard in the home impacted what they were able to discuss during a telemedicine visit. These interview results add further context to similar concerns noted in recent survey data [22]. While measures such as the use of headphones and chat features have previously been suggested as adaptations to ensure confidentiality [1, 15], our qualitative analysis suggests that these are not sufficient to ensure confidentiality of one-on-one conversations between AYAs and their healthcare team. These findings support prior literature cautioning providers on the limitations of confidentiality and related quality clinical history in telemedicine visits [23–25]. Additional research is needed about strategies for better preserving confidentiality in telehealth visits.

While a few study participants mentioned the potential of telemedicine to increase AYA patient autonomy which has previously been noted in the literature [15], participants also expressed concern about decreased patient or parent engagement during telemedicine visits. Several patients reported decreased parental engagement due to distractions by other activities during video visits. This supports research on telemedicine across multiple fields that notes challenges with patient distractions during appointments. In fact, several AYA patients expressed a preference for in-person visits as they engender more parental engagement in their healthcare. This supports a wider body of literature that shows that AYAs benefit from communication with parents about their health and parental involvement in their health and health care decisions [26–30]. Similarly, some parents emphasized the critical need for access to in-person care, reporting that some AYA patients take visits more seriously and engage more in their own care when being seen in-person. This finding supports research showing that clinicians also note that in person care may improve clinical engagement for select pediatric patients [23, 31], as well as concerns of decreased family engagement and patient accountability in the treatment of eating disorders [32]. Just as the balance of parental involvement and patient autonomy was a critical component of AYA care prior to the pandemic, our study findings emphasize that striking this balance is an ongoing goal in offering different modalities of care to patients and families. Additional research might further identify patients and families that particularly benefit from in person visits.

The benefit of telemedicine expanding access to AYA-specific services was acknowledged by study participants, particularly those with transportation or financial barriers to travel to the clinic. This is supported by our quantitative data analysis, which showed that patients living farther away from the clinic were more likely to utilize telemedicine compared to those within San Francisco or the Bay Area. This finding is congruent with prior research showing the benefits of telemedicine for patients in rural communities and those who must travel long distances for specialty care [33–35]. However, some patient populations were less likely to access care through telemedicine compared to others. Patients with public insurance were less likely to have utilized telemedicine compared to those with private insurance. This is consistent with prior evidence of pediatric primary care clinics having less telemedicine utilization among publicly insured patients [12, 13]. Barriers to telemedicine utilization among other populations have been identified, which to some extent overlap with barriers to healthcare access in general [36–38]. These may include limited technology access, low health or digital literacy, patient acceptance, cost, healthcare infrastructure and policy barriers [36, 37, 39, 40]. More work is needed to overcome barriers to telemedicine utilization within the AYA population in order to optimize its use as a means to improve access to care, while also taking into consideration that many patients or families may have a strong preference to be seen in clinic over telemedicine.

Although our qualitative data only included three non-English speakers, all three reported requiring assistance from their children with the technology used for telemedicine, which was not mentioned by any of the English-speaking participants. Previous research has shown a perception among some pediatric providers that families who use medical interpreter services may require in-person visits over telemedicine [31], and language is a well-established barrier to care in general [13, 41, 42]. Our study adds to the concern that access to telemedicine and quality of care delivered may be compromised for patients or families with limited English proficiency. Addressing these concerns likely requires systems-level improvements to eliminate these barriers [10, 40]. Such solutions might include easy-to-understand instructions in the family’s preferred language provided in advance of the visit, adequate availability of ancillary clinical staff to contact families by phone prior to the video visit to assist with connecting to the technology, and easy, fast access to professional medical interpreters by video chat [40, 43]. Clinician education focused on best practices in use of medical interpretation and telemedicine is another suggested intervention to improve quality of telemedicine for those with limited English proficiency [41].

While this study offers important adolescent and parent insights about telemedicine, it has several limitations. The interview participants were predominantly white, female, lived in San Francisco, and English-speaking. Recruitment of a more diverse group of participants may have provided further insight that was not uncovered in this study. Another limitation to our study is the sampling of a single clinic. While our clinic sees a diverse AYA patient population, cultural or practical differences across the country and around the world likely impact patient and family preferences related to telemedicine, as well as patterns of utilization of telemedicine during the pandemic. Of note, our quantitative data analyzed a period early in the pandemic, when the clinic’s capacity for in-person visits was still limited due to social distancing measures. Therefore, patients who utilize the clinic’s services more frequently may have been more likely to need to use telemedicine, while patients who only tend to be seen once or twice per year may have had more scheduling flexibility to make appointments in-person or on telemedicine based on their preferences. We also were not able to measure the patient populations chronic care burden, which may have provided better understanding of utilization of telemedicine according to their chronic care needs.

The timing of the data collection is also worth noting. The qualitative interviews were conducted in the second half of 2021, when many patients and families had been impacted by the Covid-19 pandemic for over 1.5 years. Future patient and parent perspectives will be helpful to determine if the results of our qualitative analysis hold up in the long-term or were particularly impacted by timing of the pandemic. A strength of this study’s qualitative data is the recruitment of patients and parents both who utilized telemedicine and those who were only being seen in the clinic. Therefore, the interviews included perspectives from participants who had first-hand experience with telemedicine, but also those who either had chosen not to use telemedicine or had not yet had the opportunity to experience a telemedicine visit.

## Conclusions

Our study shows that offering telemedicine as an adjunct modality to in-person visits has several potential benefits in AYA care. However, concerns about patient confidentiality and access to high-quality care remain among patients and parents. Further work is needed to better understand the mix of factors of an individual patient, family, and clinic setting that determine when an in-person visit may be more appropriate than telemedicine, and how to discuss the benefits and risks of telemedicine visits with AYA patients and families. Ultimately, understanding these nuances will be necessary for telemedicine to reach its full potential as a tool to expand access to AYA medicine services while maintaining overall quality of care and optimizing the clinician-patient interaction.

## Electronic supplementary material

Below is the link to the electronic supplementary material.

Supplementary Material 1

## Data Availability

The datasets used and/or analyzed during the current study are available from the corresponding author on reasonable request.

## List of abbreviations

AYA: Adolescent and Young Adult
